# Molecule upgrading metal-semiconductor buried contacts for high-performance and high-ideality single-crystal organic thin-film transistors

**DOI:** 10.1093/nsr/nwaf207

**Published:** 2025-05-22

**Authors:** Yongji Wang, Wei Deng, Xinmin Shi, Xiaobin Ren, Bingbing Li, Yuan Li, Jiansheng Jie, Xiujuan Zhang, Xiaohong Zhang

**Affiliations:** Institute of Functional Nano & Soft Materials (FUNSOM), State Key Laboratory of Bioin spired Interfacial Materials Science, Soochow University, Suzhou 215123, China; Institute of Functional Nano & Soft Materials (FUNSOM), State Key Laboratory of Bioin spired Interfacial Materials Science, Soochow University, Suzhou 215123, China; Macao Institute of Materials Science and Engineering (MIMSE) MUST-SUDA, Joint Research Center for Advanced Functional Materials, Macau University of Science and Technology Taipa, Macau 999078, China; Institute of Functional Nano & Soft Materials (FUNSOM), State Key Laboratory of Bioin spired Interfacial Materials Science, Soochow University, Suzhou 215123, China; Institute of Functional Nano & Soft Materials (FUNSOM), State Key Laboratory of Bioin spired Interfacial Materials Science, Soochow University, Suzhou 215123, China; School of Information Science and Engineering, Shandong University, Qingdao 266237, China; Institute of Functional Nano & Soft Materials (FUNSOM), State Key Laboratory of Bioin spired Interfacial Materials Science, Soochow University, Suzhou 215123, China; Institute of Functional Nano & Soft Materials (FUNSOM), State Key Laboratory of Bioin spired Interfacial Materials Science, Soochow University, Suzhou 215123, China; Institute of Functional Nano & Soft Materials (FUNSOM), State Key Laboratory of Bioin spired Interfacial Materials Science, Soochow University, Suzhou 215123, China

**Keywords:** contact interface, organic thin-film transistor, organic single-crystalline film, organic semiconductor

## Abstract

Achieving high-quality electrical contact at metal/organic semiconductor interfaces is crucial for unlocking the full potential of single-crystal organic thin-film transistors (SC OTFTs). However, the delicate nature of organic single-crystalline films (OSCFs) and the harsh metal deposition process often introduce trap states at the interface, limiting SC-OTFT performance. Here, we present a transparent electrical contact concept that leverages the *in situ* spontaneous reaction of fluorinated thiol molecules with the electrode, enhancing the buried metal/OSCF contacts. This method significantly lowers the Schottky barrier height by 73.3% and mitigates the Fermi-level pinning effect, resulting in over a 16-fold reduction in contact resistance. As a result, 2,7-dioctyl[1]benzothieno[3,2-b][1]benzothiophene ($\mathrm{C}_{8}$-BTBT) OTFTs achieve a high average reliable mobility ($\mu _\mathrm{r}$) of 13.2 $\mathrm{cm^2 V^{-1} s^{-1}}$ and a reliability factor up to 89%, surpassing previously reported values. Device simulations indicate that the concentration of tail and deep states is nearly two orders of magnitude lower than that of free states contributing to charge transport, suggesting near-ideal trap-free charge transport. These findings position our molecular contact upgrading method as a promising technology for advancing organic electronics.

## Teaser

A transparent contact strategy using fluorinated thiol molecules mitigates Fermi-level pinning, reducing contact resistance and achieving 13.2 cm^2^ V^−1^ s^−1^ mobility with 89% reliability in single-crystal organic thin-film transistors.

## INTRODUCTION

Metal-semiconductor electrical contacts are crucial components of electronic and optoelectronic devices [1–3], playing a key role in the performance and operation of semiconductor devices, especially those based on organic semiconductors [4–6]. As soft and flexible electronic materials, organic semiconductors offer significant opportunities for developing emerging flexible and wearable technologies [7–9], potentially serving applications not well addressed by conventional silicon technologies. To meet the demands of specific application domains, high-performance organic devices are essential, motivating significant efforts to explore organic single-crystalline films (OSCFs) that offer the highest performance among organic thin-film transistors (OTFTs) [10–13]. Despite their potential, the formation of high-quality metal-semiconductor contacts with OSCFs remains a significant challenge due to the delicate nature of these films.

Traditional metallization techniques, such as thermal evaporation, are frequently employed to create metal-semiconductor contacts in organic electronics. This process typically involves thermal radiation and metal atom bombardment, which can readily damage the delicate surfaces of organic semiconductors. Such damage can induce Fermi-level pinning at metal/organic semiconductor interfaces [4,14–16]. In particular, OSCFs possess desirable single-crystal interfaces; even slight damage during metallization can cause significant deviations in device operation from the ideal physical model, thereby degrading performance.

To minimize the surface damage during metallization, a metal electrode transfer method is widely employed in the fabrication of OTFTs based on OSCFs [5,14,17,18]. In this approach, metal and organic semiconductors interact via weak van der Waals (vdW) forces, avoiding the formation of gap trap states and energy barriers at the contact interfaces. Although this method successfully achieves clean and non-destructive metal/organic semiconductor contacts, it introduces a vdW gap that may add a Schottky barrier and reduce charge injection. Introducing a thin interlayer, such as a metal oxide layer or doping layer, at the metal/OSCF interfaces is an effective alternative strategy to overcome this limitation [16,19–21]. These interlayers reduce the depletion layer thickness at the contact region and enhance carrier interface transmission through contact doping. However, additional layers inevitably increase interface resistance ($R_\mathrm{int}$) and are significantly limited by instability and low precision due to dopant diffusion in the organic channel layers [20]. Thus, developing a new strategy to upgrade buried metal/OSCF contacts without increasing the contact thickness is crucial for achieving high-performance single-crystal OTFTs (SC OTFTs) with ideal electrical characteristics.

In this work, we present a novel strategy to transparently upgrade buried metal-OSCF contacts through the *in situ* chemical modification of the metal interface using fluorinated thiol molecules. This approach simultaneously lowers the Schottky barrier and fills the interfacial trap states, substantially alleviating the Fermi-level pinning effect at metal-OSCF contacts. Compared to the non-treated contacts, the molecule-upgraded metal/OSCF contact exhibits a 73.3% decrease in Schottky barrier height ($\Phi _\mathrm{SB}$) and over a 16-fold reduction in lower contact resistance ($R_\mathrm{c}$). As a result, SC OTFTs fabricated using the molecule upgrading buried contact strategy demonstrate a high average reliable mobility ($\mu _\mathrm{r}$) of 13.2 $\mathrm{cm^2 V^{-1} s^{-1}}$ with a large average reliability factor (*r*) of up to 89%, as well as a near-zero threshold voltage ($V_\mathrm{T}$) and low subthreshold swing (SS; 136 mV dec^−1^). This contact engineering approach offers a pathway to significantly improve the operational ideality and performance of organic electronics, paving the way for their broader application in advanced technologies.

## RESULTS AND DISCUSSION

**Figure 1.**
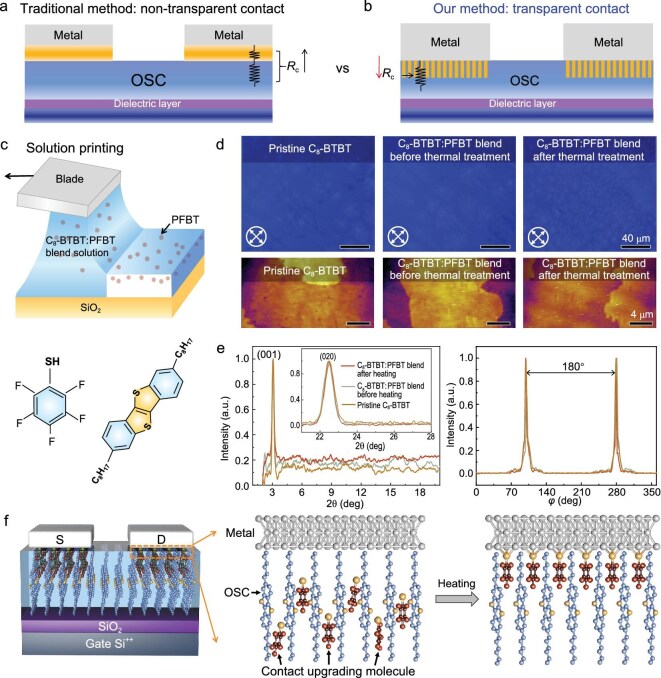
Molecule upgrading contact method. (a and b) Comparisons of the conventional interface upgrading method and our strategy. (c) Schematic illustration of the fabrication of $\mathrm{C}_{8}$-BTBT : PFBT-blend thin films by solution printing (top), and the chemical structures of PFBT and $\mathrm{C}_{8}$-BTBT molecules (bottom). (d) CPOM images and the corresponding AFM morphologies of a pristine $\mathrm{C}_{8}$-BTBT single-crystalline film and $\mathrm{C}_{8}$-BTBT : PFBT-blend thin films before and after thermal treatment. (e) Out-of-plane and in-plane XRD patterns of a pristine $\mathrm{C}_{8}$-BTBT single-crystalline film and $\mathrm{C}_{8}$-BTBT : PFBT-blend thin films before and after thermal treatment (left), and the corresponding in-plane phi-scan result of the (020) plane of these thin films (right). (f) Schematic of the process of molecule upgrading the buried contact interface in the OTFT. After thermal treatment, the PFBT molecules that resided in the $\mathrm{C}_{8}$-BTBT single-crystalline thin films migrate to the interface between the Ag electrode and $\mathrm{C}_{8}$-BTBT thin film, spontaneously reacting to form Ag–S bonds.

Introducing a thin molecular layer at the metal/organic semiconductor interface is a traditional strategy to suppress the metal-induced gap states, but the addition impedes the charge injection, leading to an increase in $R_\mathrm{c}$ (see Fig. 1a). To address this issue, we propose a transparent electrical contact concept to simultaneously alleviate interfacial trap states and reduce the $R_\mathrm{c}$, as shown in Fig. 1b. The key to the success of this method is *in situ* upgradation of the contact buried under metal electrodes. In our device, small molecules as contact upgrading materials are inserted into the alkyl chains between the $\pi$-conjugated core of the organic semiconductor (OSC) and metal layer, which avoids the unwanted charge transport disturbance. Simultaneously, these molecules can fill the interfacial trap states through charge transfer to repair the contact interface.

To implement this concept, we choose pentafluorobenzenethiol (PFBT), a typical thiol derivative, as the contact upgrading molecule because it is capable of docking to metal surfaces, forming metal-sulfur interactions that improve the work function ($W_\mathrm{F}$) of metal electrodes [22]. Moreover, the fluorine-rich functional groups within PFBT molecules act as electron donors, facilitating charge transfer and filling in-gap trap states within the band gap of organic semiconductors. Previous methods for PFBT modification that involve immersing metal surfaces in PFBT solution [8,23] are impractical for modifying buried metal-semiconductor contacts in the prevalent top-contact OTFT geometry. Considering the spontaneous reaction between PFBT and metal electrodes like Ag and Au, as well as the volatility of PFBT, we could realize an *in situ*, solvent-free procedure for modifying the buried metal-OSCF interfaces.

Initially, a blend solution of $\mathrm{C}_{8}$-BTBT and PFBT was prepared. We chose $\mathrm{C}_{8}$-BTBT as a model system due to its high mobility and stability. Employing a solution-printing technique, we fabricated $\mathrm{C}_{8}$-BTBT and PFBT-blend thin films (see Fig. 1c). Despite the introduction of additional PFBT molecules, the crystallization of $\mathrm{C}_{8}$-BTBT molecules during the solution-printing process remains unaffected due to the significantly lower concentration of PFBT relative to the $\mathrm{C}_{8}$-BTBT semiconducting material. Similarly, high-quality $\mathrm{C}_{8}$-BTBT single-crystalline films were obtained, as validated by cross-polarized optical micrography (CPOM), atomic force microscope (AFM) and X-ray diffraction (XRD) analysis. Figure 1d presents CPOM images and AFM morphologies of both a pristine $\mathrm{C}_{8}$-BTBT thin film and $\mathrm{C}_{8}$-BTBT : PFBT-blend thin films, prior to and following the thermal process. All thin films exhibit strong and uniform coloration in the CPOM image, attributable to their optical birefringence, which signifies highly crystalline structures. The progressive color shift from bright to dark, observed as the substrate is rotated relative to the polarizers’ axes, further indicates a high degree of orientation (see Figs S1–S3). AFM images reveal that $\mathrm{C}_{8}$-BTBT and PFBT-blend thin films exhibit a similar layer-like surface morphology with a step height of approximately 3.2 nm, consistent with the monolayer thickness of $\mathrm{C}_{8}$-BTBT (see Fig. S4). XRD results (see Fig. 1e) reveal no significant differences among these thin films. The pronounced (00l) peaks in the out-of-plane direction and (020) diffractions in the in-plane direction indicate that the $\mathrm{C}_{8}$-BTBT unit cells are preferentially oriented perpendicular to the substrate along the (001) axis and in-plane along the (010) axis (see Fig. 1d, left). To further verify the single-crystal nature of the PFBT-blend $\mathrm{C}_{8}$-BTBT thin films, grazing incidence XRD in-plane phi-scan measurements of the (020) plane were conducted. The appearance of two sharp peaks every 180$^{\circ }$ (see Fig. 1e, right) demonstrates the highly ordered symmetry of these thin films. Overall, the blending of PFBT with $\mathrm{C}_{8}$-BTBT does not affect the morphology or crystallinity of the resulting OSCFs.

We subsequently employed the silver as the source (S) and drain (D) contact electrodes, owing to its low melting point and relatively mild deposition conditions, which minimize damage to the single-crystal surface of the as-grown $\mathrm{C}_{8}$-BTBT thin films. The as-fabricated devices were then subjected to thermal processing in an oven, involving two steps: heating at 80 $^{\circ }$C for 60 minutes, followed by a second heating at 90 $^{\circ }$C for 90 minutes. The high volatility of PFBT facilitates its molecules within the $\mathrm{C}_{8}$-BTBT single-crystalline thin films to migrate to the buried contacts between the Ag and $\mathrm{C}_{8}$-BTBT. It is anticipated that PFBT will spontaneously react at the interface with the metal (Ag) electrode, forming thiolate-silver (Ag–S) bonds, which contribute to an increased work function of Ag. This process is illustrated in Fig. 1f. Moreover, the $\mathrm{C}_{8}$-BTBT thin film maintains its pristine single-crystal nature following the molecule upgrading contact process (see Fig. S5). This is crucial for preserving a high-quality dielectric/semiconductor interface with minimum interface trapping states, thereby ensuring an ideal carrier transport channel.

**Figure 2.**
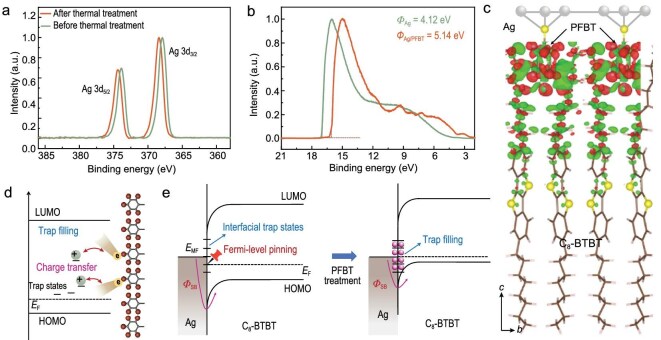
Key roles of PFBT molecules at the contact interfaces. (a) High-resolution scans of the Ag 3d XPS spectra for the pristine Ag thin film (green line) and PFBT-treated Ag electrodes (red line). (b) UPS spectra of the Ag pristine thin film (green line) and PFBT-treated Ag electrode bottom (red line). (c) Electronic density difference map between the PFBT and $\mathrm{C}_{8}$-BTBT molecules. (d) Illustration of the trap-filling mechanism. PFBT molecules become negatively charged and generate holes in the $\mathrm{C}_{8}$-BTBT thin film at the contact region to fill the trap states that are located within the band gap of $\mathrm{C}_{8}$-BTBT. (e) Schematic illustrations of the energy band diagrams of contact interfaces before and after PFBT treatment. Here $E_\mathrm{MF}$ refers to the Fermi energy level of the metal and $E_\mathrm{F}$ is the Fermi energy level of $\mathrm{C}_{8}$-BTBT.

To confirm the formation of Ag–S bonds at the base of the Ag electrode, XPS measurements were performed. Prior to this, a wet peeling-off process was developed to detach the Ag electrode from the contact region. The Ag electrode thin film was subsequently inverted to expose the underside of the treated Ag and placed onto a Si substrate (see Fig. S6). Figure 2a presents high-resolution scans of the Ag 3d XPS spectra for both pristine Ag thin films (control sample) and PFBT-treated Ag electrodes. The control sample exhibited double peaks at binding energies of 367.9 and 373.9 eV, corresponding to Ag 3d$_{5/2}$ and 3d$_{3/2}$ components of metallic Ag, respectively. Following PFBT treatment, a significant 1.5-eV increase in the binding energy of the Ag 3d peaks was observed, indicative of the reduction of Ag atoms. Additionally, the peaks at 368.4 and 374.4 eV correspond to Ag–S bonded atoms, corroborating the view that the PFBT molecules in the active layer of the OSCF interacted with silver atoms at the buried contact interfaces. Additional validation was obtained through ultraviolet photoelectron spectroscopy (UPS) measurements. The $W_\mathrm{F}$ of the Ag electrode increased from 4.12 eV for the pristine electrode to 5.14 eV following PFBT modification (see Fig. 2b). This increase supports enhanced hole injection and a reduced Schottky barrier height resulting from molecule upgrading of the buried contact.

Subsequently, density functional theory (DFT) calculations were utilized to elucidate another critical role of PFBT molecules at the contact interfaces. Based on the molecular packing structure in $\mathrm{C}_{8}$-BTBT single-crystalline films and the stable Ag-PFBT configuration [24], we constructed an interfacial contact model, wherein the PFBT molecules intercalate with the long-chain alkanes of the $\mathrm{C}_{8}$-BTBT molecules. Figure 2c displays the electronic density difference map between PFBT and $\mathrm{C}_{8}$-BTBT. It is evident that a significant charge transfer occurs between PFBT and $\mathrm{C}_{8}$-BTBT. A small quantity of electrons (0.07 a.u.) is transferred from the *p*-type $\mathrm{C}_{8}$-BTBT molecules to the fluorinated PFBT (see Fig. S7). Concurrently, an approximately 0.2-eV decrease in the S2p binding energy was observed in the PFBT-blend samples, suggesting that fluorine atoms in $\mathrm{C}_{8}$-BTBT are partially reduced in the $\mathrm{C}_{8}$-BTBT : PFBT blend (see Fig. S8). This observation corroborates the results of the DFT calculations. Building upon the DFT calculations, we propose an interpretation of the trap-filling mechanism, as illustrated in Fig. 2d. As charge transfer proceeds, PFBT molecules acquire a negative charge, thereby inducing the generation of holes in the $\mathrm{C}_{8}$-BTBT thin film at the contact region. These holes populate the trap states within the band gap of $\mathrm{C}_{8}$-BTBT, thereby healing interfacial trap states and mitigating the Fermi-level pinning effect. This mechanism is vital for attaining superior gate tunability in OTFTs. Additionally, the hole injection barrier can be reduced, leading to enhanced performance (see Fig. 2e).

**Figure 3.**
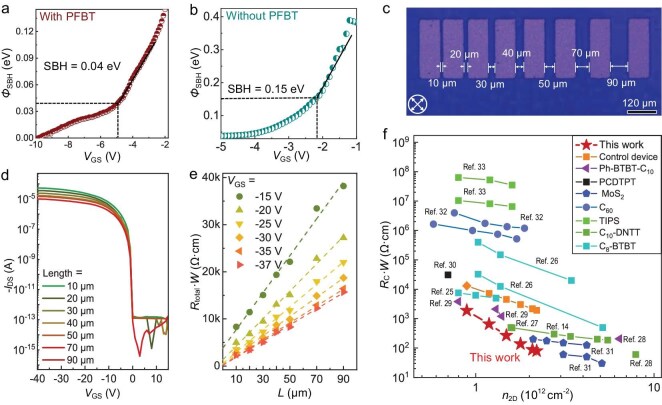
Characterization of contact interfaces. (a and b) The Schottky barrier height $\Phi _\mathrm{SB}$ as a function of $V_\mathrm{GS}$ for the Ag/$\mathrm{C}_{8}$-BTBT contact interfaces with and without PFBT treatment. (c) CPOM image of $\mathrm{C}_{8}$-BTBT single-crystalline thin-film-based devices with different *L* using the TLM. (d) Transfer characteristics of the PFBT upgrading devices with various *L* of 10, 20, 30, 40, 50, 70, 90 and 100 μm in the linear regime. (e) The total resistance $R_\mathrm{total}$ as a function of *L* at various $V_\mathrm{GS}$. The *y*-axis intercepts at zero channel length are equal to the $R_\mathrm{c}$. (f) The contact resistance $R_\mathrm{c}$ as a function of $n_\mathrm{2D}$ for our PFBT upgrading device and the previously reported devices.

To elucidate the improved contact interface in OTFTs utilizing $\mathrm{C}_{8}$-BTBT single-crystalline films, we performed variable-temperature device measurements to extract the $\Phi _\mathrm{SB}$ values using the Arrhenius equation (see Fig. S9). The $\Phi _\mathrm{SB}$ value of the control device without PFBT treatment (pristine OTFT) is 150 meV, while for the device with PFBT upgrading contacts, it decreases to as low as 40 meV (see Fig. 3a and b). The significant reduction in $\Phi _\mathrm{SB}$ corroborates the improvement in device contact quality, which is expected to similarly reduce $R_\mathrm{c}$. We further evaluated $R_\mathrm{c}$ using the transfer length method (TLM), employing $\mathrm{C}_{8}$-BTBT single-crystalline films as the channel. Figure 3c presents a CPOM image of the device with varying channel lengths (*L*) used for TLM analysis. We investigated the transfer characteristics of the devices with PFBT upgrading devices, measured in the linear regime (see Fig. 3d). Figure 3e depicts the width-normalized total resistance ($R_{\mathrm{total}}W$) as a function of channel length (*L*) within a gate voltage ($V_\mathrm{GS}$) range of $-15$ to $-37$ V. The $R_\mathrm{c}$ values were extracted from the zero-length intercept of the linear fit of $R_{\mathrm{total}}W$. The minimum $R_\mathrm{c}$ was found to be 79.7 $\Omega$ cm at $V_\mathrm{GS} = -37$ V, corresponding to a carrier concentration ($n_\mathrm{2D}$) of $2.2\times 10^{12}\mathrm{cm^{-2}}$. To the best of our knowledge, this $R_\mathrm{c}$ value is approximately 2.5 times lower than that of previously reported OTFTs at the same $n_\mathrm{2D}$ (see Fig. 3f and Table S1) [14,25–33]. In contrast, for control devices lacking a PFBT upgrading contact interface, the extracted $R_\mathrm{c}$ value is 1314 $\Omega$ cm at the same $n_\mathrm{2D}$ (see Fig. 3f and Fig. S10), which is approximately 16.5 times larger than that of the PFBT upgrading contact. This finding underscores the high quality of the metal/organic semiconductor buried contact achieved through PFBT treatment.

**Figure 4.**
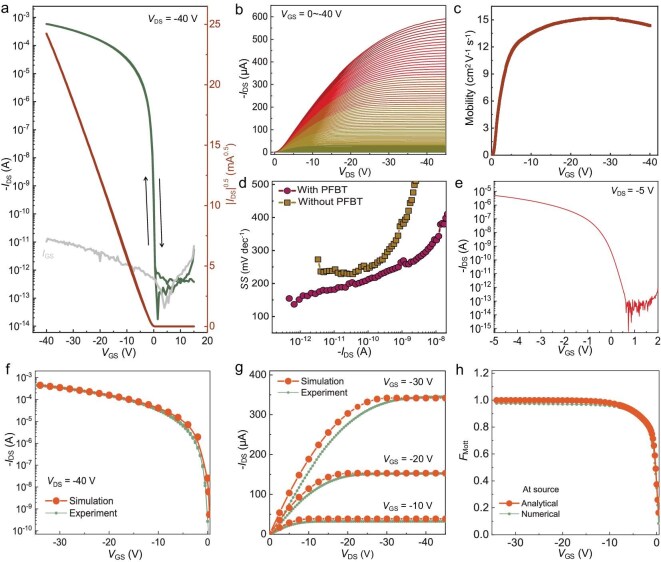
Electrical properties of the $\mathrm{C}_{8}$-BTBT SC OTFT with PFBT upgrading contact. (a) Typical transfer characteristic and (b) the corresponding output characteristic of the SC OTFT. (c) Saturation mobility as a function of $V_\mathrm{GS}$. (d) SS as a function of $I_\mathrm{DS}$ for the SC OTFTs with and without PFBT upgrading contact. (e) Transfer characteristic for the PFBT upgrading SC OTFT under a low $V_\mathrm{DS}$ of $-5$ V. Comparison of (f) transfer and (g) output characteristics of the PFBT upgrading OTFTs between the simulation and experimental results. (h) Analytical and numerical results of $F_\mathrm{Mott}$ at source of the devices as a function of $V_\mathrm{GS}$.

The electrical properties of $\mathrm{C}_{8}$-BTBT single crystalline film-based OTFTs with PFBT-upgraded contact were systematically characterized. The SC OTFT demonstrates ideal transfer and output characteristics, including a nearly zero $V_\mathrm{T}$ of $-0.47$ V, an impressive on : off ratio exceeding $10^{10}$, negligible hysteresis and impressive leakage current ($I_\mathrm{GS}$) (see Fig. 4a and b). The saturation mobility, derived from the slope of the transfer curve, is 15.3 $\mathrm{cm^2\ V^{-1} s^{-1}}$, which is comparable to the linear mobility (see Fig. S11). At elevated $V_\mathrm{GS}$ ranges, the saturation mobility remains independent of the gate voltage (see Fig. 4c), affirming the reliability of the calculated mobility value. In accordance with Podzorov’s guidelines [21], we calculated $\mu _\mathrm{r}$ and *r* of the device (see Fig. S12). Our device with PFBT upgrading contacts exhibited a high $\mu _\mathrm{r}$ of 14.5 $\mathrm{cm^2\ V^{-1} s^{-1}}$ with a very large *r* of 95%, approaching 100%, which validates the high ideality of our SC OTFT. Furthermore, the PFBT-upgraded contact remained stable even after prolonged bias voltage stress (see Fig. S13), underscoring the robustness of the contact interface, which can be ascribed to the chemical bonding between Ag atoms and PFBT molecules.

The SS is a critical parameter for OTFTs, as it is influenced by the interfacial state density at both the dielectric-semiconductor interface and the metal-semiconductor contact [8]. Figure 4d presents the extracted SS values for various drain currents ($I_\mathrm{GS}$). The minimum SS extracted from the transfer curve is as low as 136 mV dec^−1^, indicating that our device exhibits exceptional gate tunability. In contrast, the control device displays a significantly higher SS value of 237 mV dec^−1^, indicating reduced gate modulation efficiency. Following PFBT modification of the contact, we observed a substantial reduction in the SS, which signifies an enhanced gate-induced charge injection barrier-lowering effect. This constitutes strong evidence of the alleviation of Fermi-level pinning. Significantly, due to the extremely low SS, our $\mathrm{C}_{8}$-BTBT SC OTFT operates effectively at a low supply voltage of $-5$ V, while maintaining a high on : off ratio exceeding 10$^{8}$ (see Fig. 4e). This result suggests that our contact upgrading method significantly reduces power dissipation in OTFTs, rendering it highly promising for large-area wearable electronics and the internet of things.

According to trap-and-release transport theory, charge carriers in semiconductors originate from both delocalized and localized electronic states, which are separated by the mobility edge ($E_\mathrm{m}$). The delocalized states above $E_\mathrm{m}$ facilitate the transport of free carriers. The localized states below $E_\mathrm{m}$ include tail states (shallow traps) and deep states (deep traps), which are responsible for trapping and releasing free carriers. To quantitatively analyze these states, we conducted device simulations using a recently developed generic model for thin-film transistors [34]. Through reproducing the transfer and output characteristics of the SC OTFT (see Fig. 4f and g), simulations revealed that the concentration of free states contributing to charge transport in the $\mathrm{C}_{8}$-BTBT thin films is approximately $1.0\times 10^{18}\mathrm{cm^{-3}}$, and that the concentrations of tail and deep states that limit charge transport are $4.0\times 10^{16}$ and $1.5\times 10^{16}\mathrm{cm^{-3}}$, respectively. Notably, the *n*_tail_ and *n*_deep_ are comparable to those found in a range of organic single-crystal materials, including rubrene (10$^{15}$–10$^{17}\mathrm{cm^{-3}}$) [35], dibenzo-tetrathiafulvalene (10$^{16}$)[36] and pentacene (10$^{15}$) [37]. Since the concentration of trap states is significantly lower than that of free states in the device, the ratio of free charge $q_\mathrm{free}$ to total charge $q_\mathrm{total}$ in the device channel, i.e., the so-called Mott function ($F_\mathrm{Mott} = q_\mathrm{free}/q_\mathrm{total}$) [34], approaches almost 100% when the device is fully turned on (see Fig. 4h). These results demonstrate nearly ideal trap-free carrier transport in the PFBT-upgraded SC OTFT. Furthermore, the simulated characteristics of our SC OTFT align closely with the experimental data, further confirming that the contact interface after PFBT molecule upgrading has indeed achieved the ideal physical model. Our contact upgrading method is also applicable to other OSCs, including the *n*-type small-molecule semiconductor and polymer semiconductor. The performance of both *n*-type and *p*-type devices was improved to some extent after employing our method, witnessing the universality of our method (see Fig. S14).

**Figure 5.**
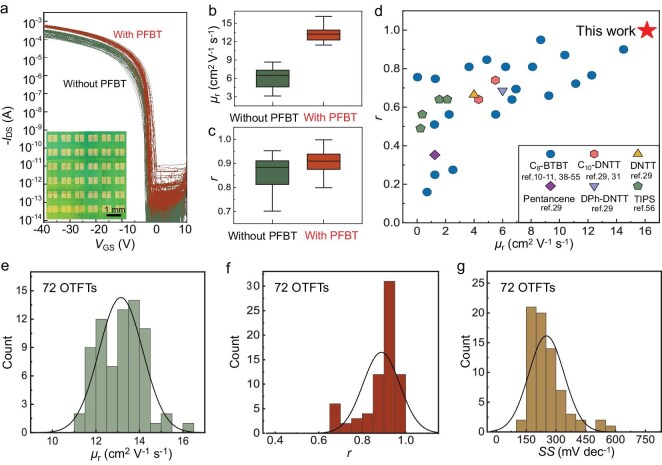
Statistical analysis on the PFBT upgrading SC OTFTs and comparison with literature data. (a) Transfer curves of the PFBT upgrading SC OTFTs (red) and control devices (green). (b and c) The reliable mobility $\mu _\mathrm{r}$ and *r* plotted as box plots between the PFBT upgrading SC OTFTs and control devices. (d) Comparison of the average $\mu _\mathrm{r}$ versus *r* for the previously reported OTFTs and our PFBT upgrading SC OTFTs. (e–g) Statistical histograms of $\mu _\mathrm{r}$, *r* and the SS of the PFBT upgrading SC OTFTs.

To assess the scalability of our strategy, we fabricated arrays of $\mathrm{C}_{8}$-BTBT SC OTFTs both with and without PFBT treatment. We randomly selected 72 devices from the central regions of two independent sample batches (36 devices per batch) to ensure spatial uniformity and to minimize edge effects. Figure 5a illustrates the differences in transfer curves between the PFBT upgrading SC OTFTs and control devices. The $\mu _\mathrm{r}$ value for both types of SC OTFT are plotted in Fig. 5b, offering a clearer representation of their differences. Clearly, the average $\mu _\mathrm{r}$ value of the PFBT upgrading SC OTFTs is 13.2 $\mathrm{cm^2\ V^{-1} s^{-1}}$, which is nearly 2.2 times higher than that of the control devices (6 $\mathrm{cm^2\ V^{-1} s^{-1}}$), and the maximum $\mu _\mathrm{r}$ is 16.1 $\mathrm{cm^2\ V^{-1} s^{-1}}$. Thanks to the high-quality contact interface, all SC OTFTs also exhibit ideal electrical characteristics. Among these, 86% of the devices achieved an *r* value exceeding 80% with an average *r* ($r_\mathrm{avg}$) of approximately 89% (see Fig. 5c). These $\mu _\mathrm{r}$ and *r* values are significantly higher than those reported previously (see Fig. 5d and Table S2) [10,11,29,31,38–56], achieving both high mobility and ideality in OTFTs for the first time, to our knowledge. Panels (e)–(g) of Fig. 5 present corresponding histograms for $\mu _\mathrm{r}$, *r* and the SS, respectively. The coefficients of variation for $\mu _\mathrm{r}$, *r* and the SS were found to be 7.6%, 9.4% and 34.8%, respectively. The $V_\mathrm{T}$ exhibited a distribution of $-0.35\pm 0.19$ V (see Fig. S15), a deviation of only 0.475% of the operating gate-source voltage window ($-40$ V). Notably, the mobility coefficient of variation is significantly lower than that of the control devices (26.7%), underscoring the large-area uniformity of our SC OTFTs following PFBT upgrading. This uniform device performance can be attributed to the robustness of our contact upgrading method, as well as the large-area single-crystalline nature of the $\mathrm{C}_{8}$-BTBT thin films. The consistent and high-performance metrics across a large number of devices highlight the effectiveness and scalability of the PFBT upgrading strategy. This approach holds significant promise for the development of high-performance, large-area electronics and wearable devices, owing to its ability to enhance both mobility and reliability in OTFTs.

## CONCLUSION

In conclusion, we have proposed a novel strategy for upgrading a metal/organic semiconductor contact interface that simultaneously enhances both the performance and ideality of SC OTFTs. This strategy utilizes the spontaneous reaction between metal electrodes and fluorinated thiol molecules to enhance buried contacts in SC OTFTs, effectively reducing the Schottky barrier and filling interfacial trap states. Our approach leads to a 73.3% reduction in $\Phi _\mathrm{SB}$ and achieves a notably low $R_\mathrm{c}$ of $79.7\Omega$ cm, approximately 2.5 times lower than previously reported OTFTs at the same $n_\mathrm{2D}$. Thanks to these enhanced contacts, $\mathrm{C}_{8}$-BTBT SC OTFTs achieve an impressive average $\mu _\mathrm{r}$ of 13.2 $\mathrm{cm^2\ V^{-1} s^{-1}}$ and an unprecedented large *r* exceeding 89% simultaneously. These metrics are significantly higher than those of previously reported OTFTs. Moreover, SC OTFTs with PFBT-upgraded contacts exhibit lower SS and reduced $V_\mathrm{T}$, compared to control devices, further confirming the superior quality of the contact interface. We believe that our buried contact upgrade method represents a pivotal breakthrough in achieving high-performance and high-ideality OTFTs, facilitating the development of more advanced and higher-performing organic electronic devices.

## MATERIALS AND METHODS

### Materials

The $\mathrm{C}_{8}$-BTBT utilized in this study was purchased from Luminescence Technology Corp. Anhydrous toluene, serving as the solvent, was sourced from Shanghai Titan Scientific Co. Ltd. Polystyrene (PS; $M_\mathrm{W} = 2000$ kDa) and PFBT were obtained from Sigma-Aldrich. All materials were used as received. Highly doped *n*-type silicon wafers with a resistivity of approximately 0.01 $\Omega$ cm were employed as substrates. These silicon wafers, obtained from SUMCO Corp., featured a 300-nm thermally grown silicon oxide dielectric layer.

### Fabrication and characterizations of $\mathbf{C}_{\mathbf 8}$-BTBT : PFBT-blend thin films

To enhance the quality and uniformity of $\mathrm{C}_{8}$-BTBT thin films, PS was incorporated as a binder. A solution containing $\mathrm{C}_{8}$-BTBT, PS and PFBT was prepared in toluene with a fixed weight ratio of 5 : 10 : 2.97, maintaining a $\mathrm{C}_{8}$-BTBT concentration of 5 mg mL^−1^. The use of PS is intended to improve the crystallinity of $\mathrm{C}_{8}$-BTBT thin films and passivate defects at the dielectric-OSC interface. The optimal weight percentage of PFBT was determined through controlled experiments (see Fig. S16). Prior to use, the blend solution was stirred for 3 hours to ensure complete dissolution. The growth of $\mathrm{C}_{8}$-BTBT OSCFs was carried out in a homebuilt blade-coating setup. An approximately 1.5 μL mixed solution was injected into the gap between the substrate and Si blade. The blade angle was set at approximately 15$^{\circ }$ and a gap distance between the blade and the substrate was controlled at around 100 μm. Then, the blade was controlled by a stepper motor at a speed of 200 μm s^−1^ at room temperature in air. Following film formation, morphology was assessed using a CPOM (Olympus BX51) and AFM (Asylum Cypher S). XRD measurements were performed with a Bruker D8 DISCOVER X-ray diffractometer. Out-of-plane data were collected in 2$\theta /\Omega$ mode, while in-plane data were obtained using a grazing incidence with $\omega$ = 0.15$^{\circ }$ and 2$\theta \chi /\Phi$ scan mode, capturing (020) and (200) peaks with angles of 2$\theta$ = 0.15$^{\circ }$ and 3$^{\circ }$, respectively.

### OTFT fabrication and measurement

Source/drain electrodes (40-nm Ag) were deposited by vacuum evaporation through a shadow mask at a rate of 0.4 Å s^−1^. Channels *L* and ${\it W}$ were defined as 60 and 480 μm, respectively. The deposition was conducted at a chamber pressure lower than $5\times 10^{-5}$ Torr. The capacitance of the bilayer gate dielectric (PS and $\mathrm{SiO_2}$) was measured to be 6.3 nF cm$^{-2}$ (see Fig. S17). The devices were subsequently placed in a vacuum oven (Memmert VO 200) and heated at $80^{\circ }$C for 60 minutes, followed by a second heating at $90^{\circ }$C for 90 minutes after a rest period. The electrical properties of the OTFTs were measured using a probe station connected to an Agilent B1500A semiconductor analyser. Before electrical measurements, all devices were patterned by mechanical scratches to avoid the cross-talk effects. The variable-temperature measurements of the OTFTs were conducted in the dark using a cryogenic probe station at $10^{-4}$ Torr.

## Supplementary Material

nwaf207_Supplemental_File
