# Supplementary material for: Molecule upgrading metal-semiconductor buried contacts for high-performance and high-ideality single-crystal organic thin-film transistors
Source: Natl Sci Rev. 2025 May 22;12(7):nwaf207. doi: 10.1093/nsr/nwaf207 (PMC12163995; doi:10.1093/nsr/nwaf207)
Supplement: nwaf207_Supplemental_File [file nwaf207_supplemental_file.pdf]

Supporting Information

Molecule upgrading metal-semiconductor buried contacts for high-performance and high-ideality single-crystal organic thin-film transistors

Yongji Wang[1], Wei Deng[1,], Xinmin Shi[2], Xiaobin Ren[1], Bingbing Li[1], Yuan Li[3], Jiansheng Jie[1], Xiujuan Zhang[1,*], and Xiaohong Zhang[1,*]*

[1]Institute of Functional Nano & Soft Materials (FUNSOM)
Jiangsu Key Laboratory for Carbon-Based Functional Materials & Devices
Soochow University
Suzhou, Jiangsu 215123, China

[2]Macao Institute of Materials Science and Engineering (MIMSE)
MUST-SUDA Joint Research Center for Advanced Functional Materials
Macau University of Science and Technology
Taipa, Macau SAR 999078, China

[3]School of Information Science and Engineering
Shandong University
Qingdao, 266237, China

***Corresponding authors.** *Email: dengwei@suda.edu.cn; xjzhang@suda.edu.cn; xiaohong_zhang@suda.edu.cn*

This file includes:

Methods (Page 2-5)
Supplementary Figures 1 to 17 (Page 6-23)
Supplementary Tables 1 to 2 (Page 24-25)

Methods

XPS and UPS measurements

For X-ray photoelectron spectroscopy (XPS) and ultraviolet photoelectron spectroscopy (UPS) sample preparation, a wet peeling-off process was developed. Initially, a C_8-BTBT: PFBT blend thin film was grown on the SiO_2/Si substrates followed by thermal evaporation of a 150 nm silver film. The sample was then subjected to thermal processing in an oven at 80°C for 60 minutes and 90°C for 90 minutes. Afterward, the sample was immersed in toluene solvent to detach the Ag thin film which was subsequently flipped and transferred to a Si wafer for XPS and UPS analysis. XPS spectra were recorded using a PHI Genesis (XPS) Surface Analysis Instrument with a base pressure 2.5×10^{-10} Torr, employing an Al Kα monochromatic source and a Spherical Capacitor Analyzer with a 0.8 mm diameter slit aperture. C1s spectra were referenced to the C-C/C-H binding signal at 285.0 eV. UPS measurements were performed in a UHV UPS system (pressure < 5×10^{-10} Torr), utilizing He I (hv = 21.2 eV) photon excitation. Satellite line features from the He discharge lamp were subtracted from each UPS spectrum.

DFT calculations

The DFT calculations were performed using the Vienna Ab initio Simulation Package (VASP) with the projector augmented wave (PAW) method. To focus on the charge transfer mechanism between PFBT and C_8-BTBT, the interaction between Ag and C_8-BTBT was intentionally omitted in the computational model. This simplification aligns with experimental observations (XRD and AFM), which confirmed that PFBT resides within the alkyl side chains of C_8-BTBT without disrupting its single-crystalline lattice. A vacuum layer of ≥15 Å was introduced between the Ag substrate and the C_8-BTBT layer to eliminate spurious interactions.

The Perdew-Burke-Ernzerhof (PBE) functional under the generalized gradient approximation (GGA) was employed for geometry optimization and electronic structure analysis. The PBE functional is widely validated for organic-metal interfaces

due to its balance between computational efficiency and accuracy in predicting charge redistribution trends. A plane-wave cutoff energy of 900 eV and a Γ-centered 3 × 3 × 1 k-point grid ensured convergence of total energy and forces (<0.01 eV/Å). While PBE may underestimate bandgaps, this approach suffices for qualitative analysis of interfacial charge transfer, as supported by experimental S2p binding energy shifts and UPS-derived work function modifications.

Extraction of Schottky barrier height

The Φ_{SB} was extracted from the transfer characteristics of devices recorded at 195-270 K. The drain current density (I_{DS}) thermally injected from the metal contact into the channel through a Schottky barrier can be expressed as:

$$I_{DS} = AA^{*}T^{2}\, exp\,(-\frac{\Phi_B}{k_B T})[1 - exp\,(\frac{-V_{DS}}{k_B T})] \quad (1)$$

where A is the junction area, A^* is the Richardson constant, T is the temperature, k_B is Boltzmann's constant, q is the elementary charge, and Φ_B is the effective contact barrier height at a given gate–source voltage (V_{GS}). If $V_{DS} \gg k_B T$, equation (1) can be simplified to

$$I_{DS} \approx AA^{*}T^{2}\, exp\left(-\frac{\Phi_B}{k_B T}\right) \quad (2)$$

In this case, the effective energy barrier at a given V_{GS} can be extracted by finding the slope in the Arrhenius plots, as shown in Fig. S7, Supporting Information, using the following equation:

$$ln\,(I_{DS}/T^{2}) = \frac{-\Phi_B}{k_B T} + c \quad (3)$$

In the subthreshold regime, the barrier decreased linearly. When the V_{GS} is above a certain value, the barrier saturates and decreased nonlinearly. The ordinate of the changing point corresponds to the Schottky barrier height.

Extraction of R_C

Total device resistance (R_{total}, in units of Ω cm) normalized by channel width (W) can be expressed as:

$$R_{total} \cdot W = R_{sheet} \cdot L + 2R_c \cdot W \quad (4)$$

where R_{sheet} is the sheet resistance of the semiconductor channel. The R_{total} varies linearly with the L if R_c and R_{sheet} are spatially homogeneous in the device. Therefore, by measuring the total resistances of the devices with various L, the R_{total} can be plotted as a function of L. The residual resistance at L = 0 corresponds to the total contact resistance ($2R_c$) of the device.

Calculation of reliable mobility and reliability factor

The reliable mobility, μ_r, is recently proposed to prevent the OTFT performance from being overestimated. The μ_r was evaluated using the following formulas:

$$\mu_{sat} = (\frac{2L}{WC_i})(\frac{\partial\sqrt{|I_{DS}|}}{\partial V_G})^2 \tag{5}$$

The μ_r is:

$$\mu_r = (\frac{2L}{WC_i})(\frac{\sqrt{|I_{DS}|^{max}}-\sqrt{|I_{DS}|^0}}{|V_{GS}|^{max}})^2 \tag{6}$$

The reliability factor, r, reflects the operation ideality of OTFTs, it can be expressed as:

$$\mu_r = r \times \mu_{sat} \tag{7}$$

Device Simulations

The concentration of charge carriers accumulated in the device channel for free/tail/deep states reads:

$$n_{\mathrm{free/tail/deep}} = N_{\mathrm{free/tail/deep}} \exp\left(\frac{E_{\mathrm{VBM}}-E_{\mathrm{F}}}{\varphi_{\mathrm{free/tail/deep}}}\right) \tag{8}$$

Where $N_{free/tail/deep}$ and $\varphi_{free/tail/deep}$ denote the effective carrier concentration at the valence band maximum (E_{VBM}) of C_8-BTBT crystal and characteristic potential of free/tail/deep states, respectively, and E_F represents the Fermi level. The electrostatic potential (φ) distributed in the channel can be obtained by solving the Poisson equation:

$$\frac{d^2\varphi}{dx^2} = -\frac{q}{\varepsilon_s}\left(n_{\mathrm{free}} + n_{\mathrm{tail}} + n_{\mathrm{deep}}\right) \tag{9}$$

Where ε_s denotes the permittivity of C_8-BTBT and x is perpendicular to the transport direction. Assuming that only free carriers contribute to charge transport, the drain

current I_{DS} can be obtained by the drift-diffusion equation:

$$I_{\mathrm{DS}} =- W\mu_{\mathrm{free}}\left(q_{\mathrm{free}}\frac{\mathrm{d}\varphi_{\mathrm{s}}}{\mathrm{d}y}+\varphi_{\mathrm{free}}\frac{\mathrm{d}q_{\mathrm{free}}}{\mathrm{d}y}\right) \tag{10}$$

where W denotes the channel width, μ_{free} = 15 cm^{2}V^{-1}s^{-1} the free-carrier mobility, $q_{\mathrm{free}} =\int_{x=0}^{x=T_S} qn_{\mathrm{free}}\mathrm{d}x$ the free charge density (with T_S the channel thickness), φ_s the surface potential at the dielectric/semiconductor interface, and y the transport direction.

The Mott function, defined as $F_{\mathrm{Moot}} = q_{\mathrm{free}}/q_{\mathrm{total}}$ with $q_{\mathrm{total}} =\int_0^{T_S} q(n_{\mathrm{free}} + n_{\mathrm{tail}} + n_{\mathrm{deep}})\mathrm{d}x$, can also be expressed approximately as:

$$F_{\mathrm{Mott}} = \left\{1+\frac{N_{\mathrm{deep}}}{N_{\mathrm{free}}}\exp\left[a_{\mathrm{deep}}\left(b_{\mathrm{deep}}-V_{\mathrm{GS}}\right)\right]+\frac{N_{\mathrm{tail}}}{N_{\mathrm{free}}}\exp\left[a_{\mathrm{tail}}\left(b_{\mathrm{tail}}-V_{\mathrm{GS}}\right)\right]\right\}^{-1} \tag{11}$$

where $a_{\mathrm{deep/tail}}$ and $b_{\mathrm{deep/tail}}$ denote fitting parameters associated with the deep/tail states.

Supporting figures

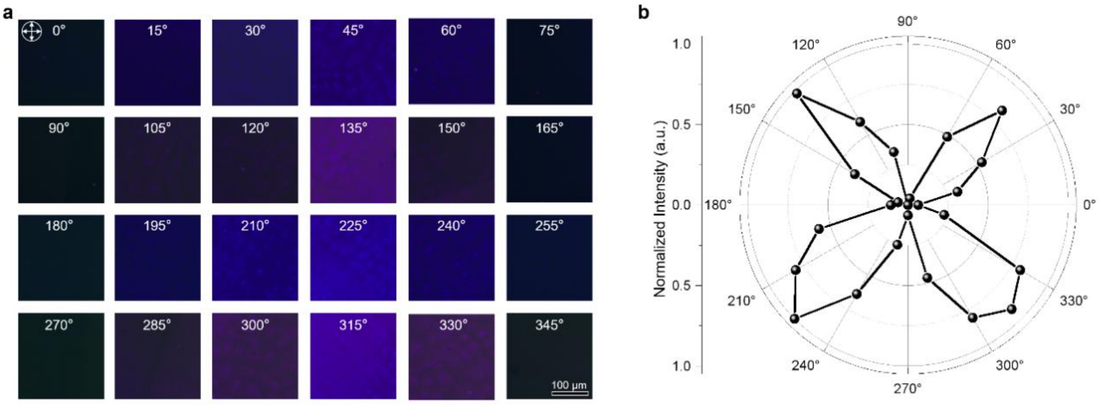

Figure S1. Orientation characterization of pristine C_8-BTBT thin films. (a) CPOM images of pristine C_8-BTBT thin films captured under different polarization angles from 0° to 360°. (b) Normalized intensity of the C_8-BTBT thin film under CPOM as a function of polarization angle.

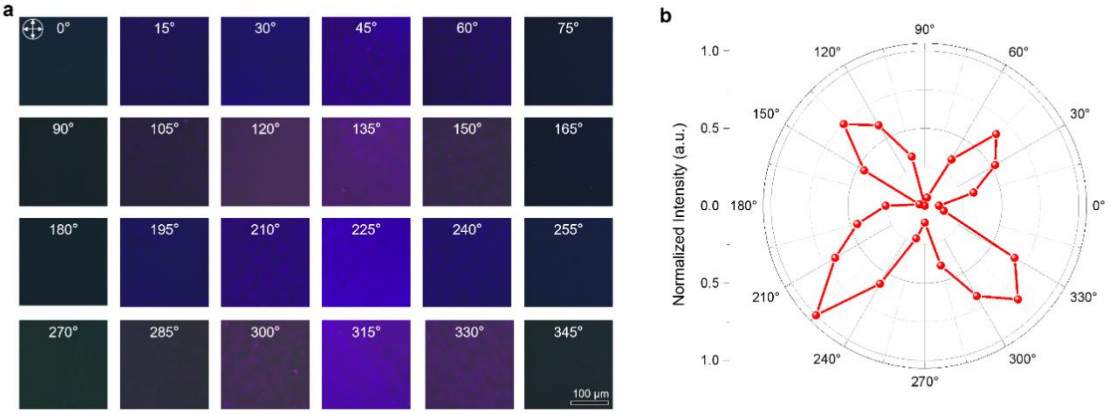

Figure S2. Orientation characterization of C_8-BTBT: PFBT blend thin films before thermal treatment. (a) CPOM images of C_8-BTBT: PFBT blend thin film captured under different polarization angles from 0° to 360°. (b) Normalized intensity of the C_8-BTBT: PFBT blend thin film under CPOM as a function of polarization angle.

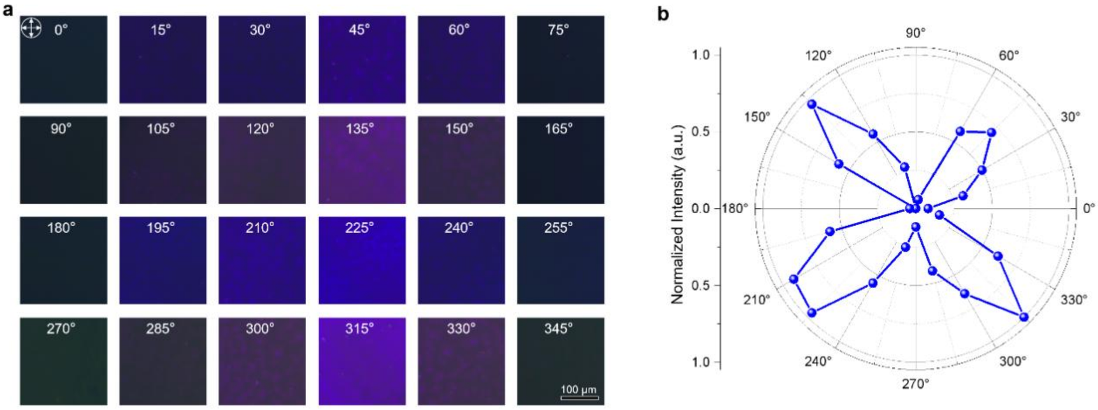

Figure S3. Orientation characterization of C_8-BTBT: PFBT blend thin films after thermal treatment. (a) CPOM images of C_8-BTBT: PFBT blend thin film captured under different polarization angles from 0° to 360°. (b) Normalized intensity of the C_8-BTBT: PFBT blend thin film under CPOM as a function of polarization angle.

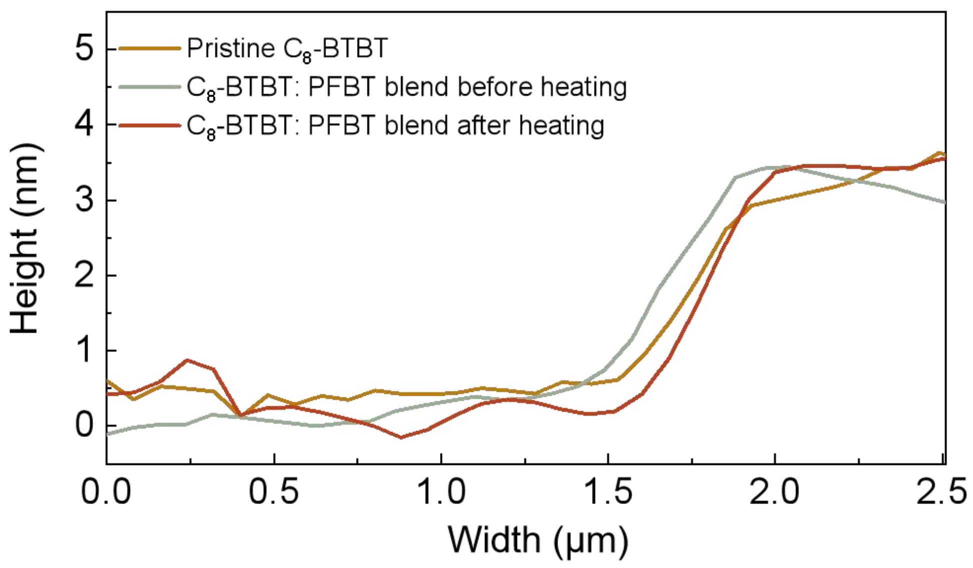

Figure S4. Height profiles of the layer-like surfaces on the pristine C_8-BTBT single-crystalline film and C_8-BTBT: PFBT blend thin films before and after thermal treatment.

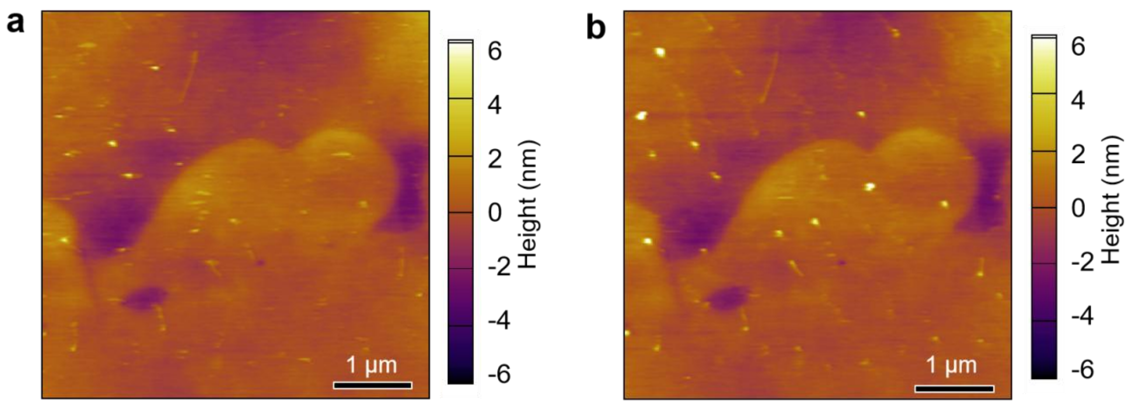

Figure S5. Morphologies of C_8-BTBT: PFBT blend thin films in the channel region before (a) and after (b) thermal treatment.

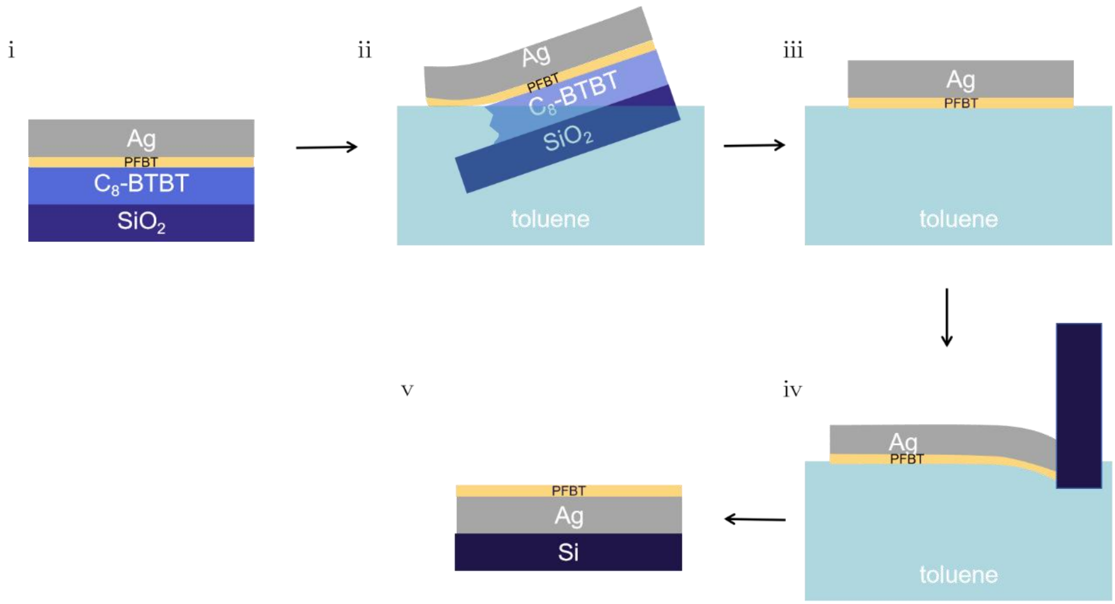

Figure S6. Schematic of the wet peeling-off process to expose the PFBT-treated Ag bottom surface for XPS measurements.

To dissolve the underlying organic layer using toluene, which caused the electrode thin film to detach and float on the liquid surface and the floating electrode film was then carefully flipped over onto a silicon substrate. This flipping process exposed the bottom surface of the electrodes, which was originally in contact with the organic layer. So that the exposed bottom surface was then directly accessible for XPS analysis.

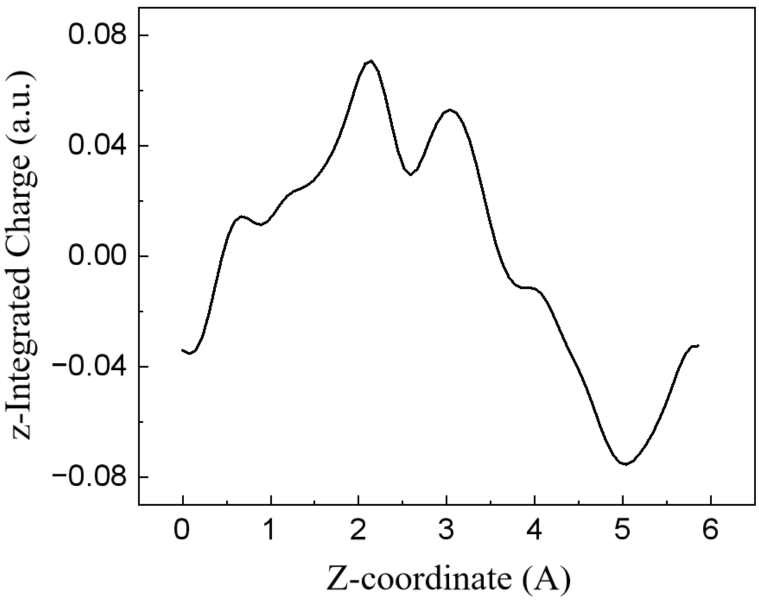

Figure S7. Density difference curve of C_8-BTBT and PFBT system. It is integrated on (x, y) planes and plotted along the z direction.

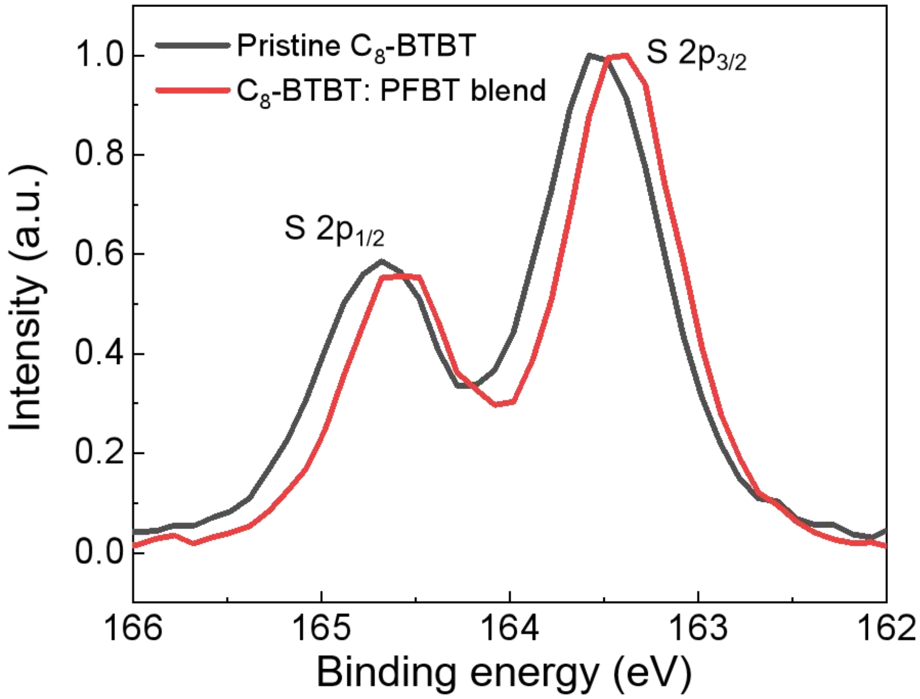

Figure S8. High-resolution scans of the S 2p XPS spectra for the pristine C_8-BTBT single-crystalline film and C_8-BTBT: PFBT blend single-crystalline film.

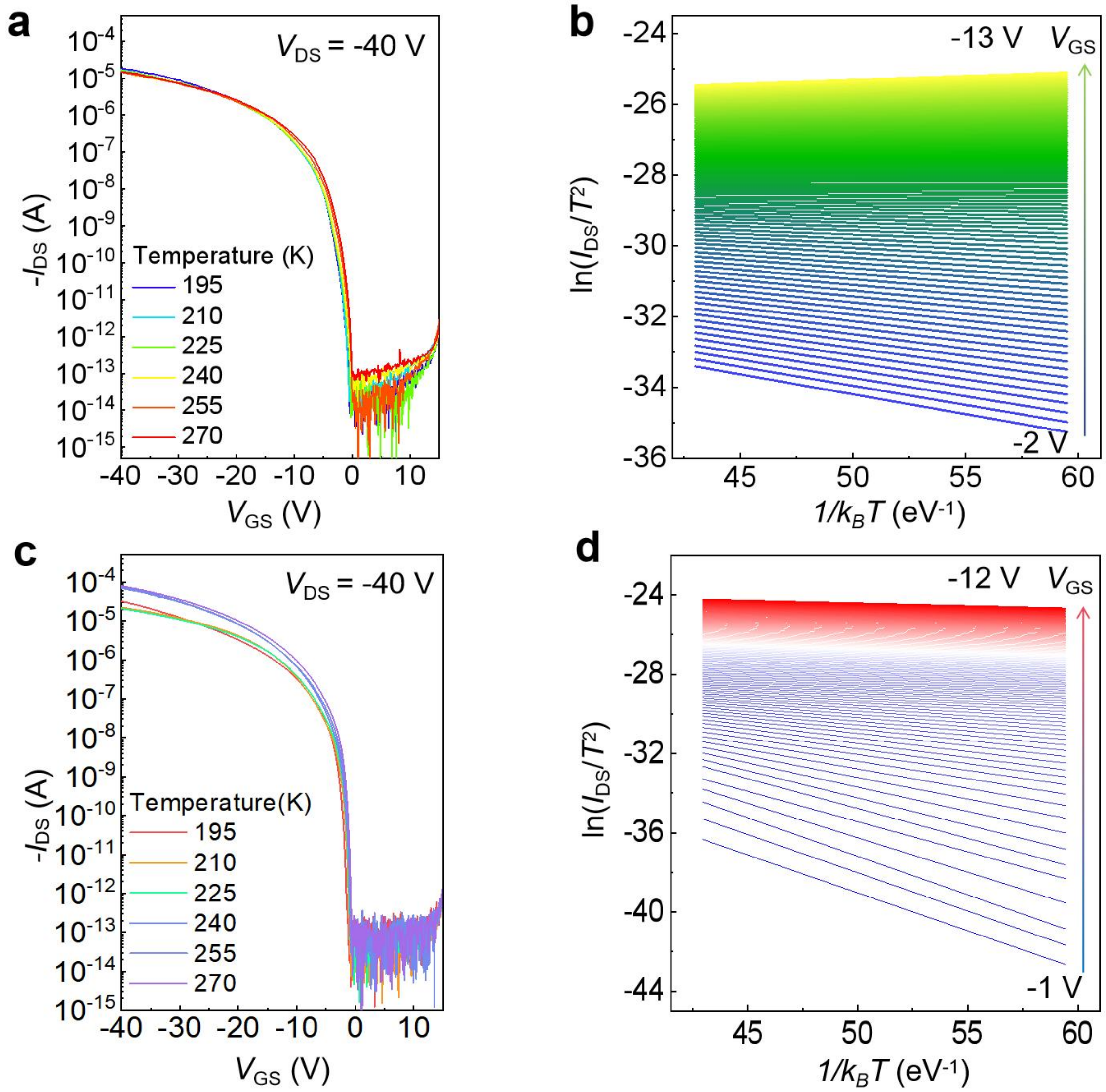

Figure S9. Temperature-dependent transfer characteristics of the OTFTs with (a) and without PFBT treatment (c). (b, d) The corresponding linear fit of the Arrhenius plot of $\ln(I_{DS}/T^2)$ versus $1/k_BT$ for the devices.

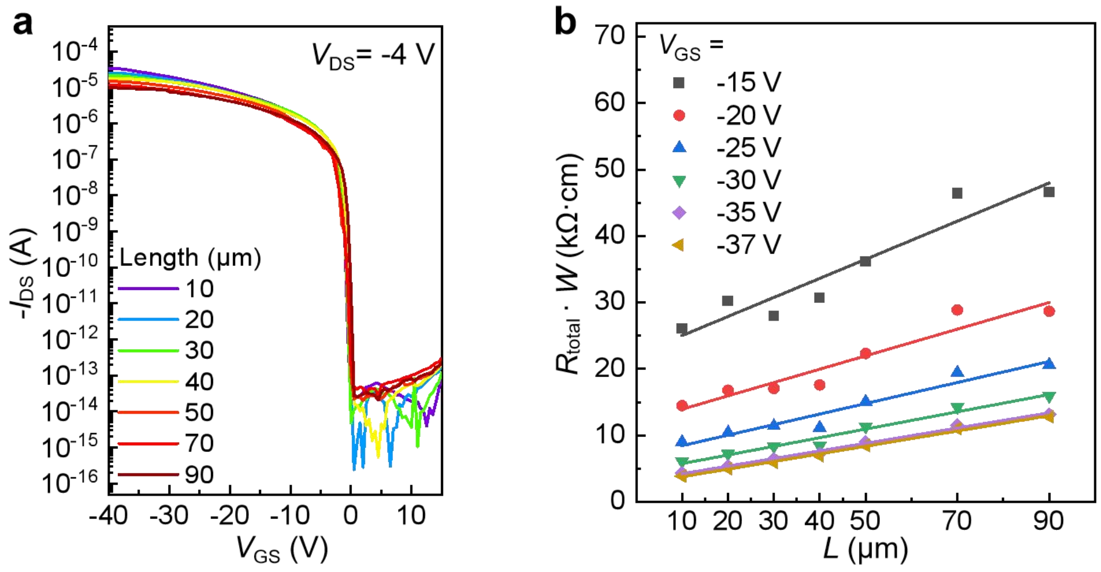

Figure S10. (a) Transfer characteristics of the control devices without PFBT upgrading contact interface. b) $R_{\text{total}} \cdot W$ as a function of L at various V_{GS}.

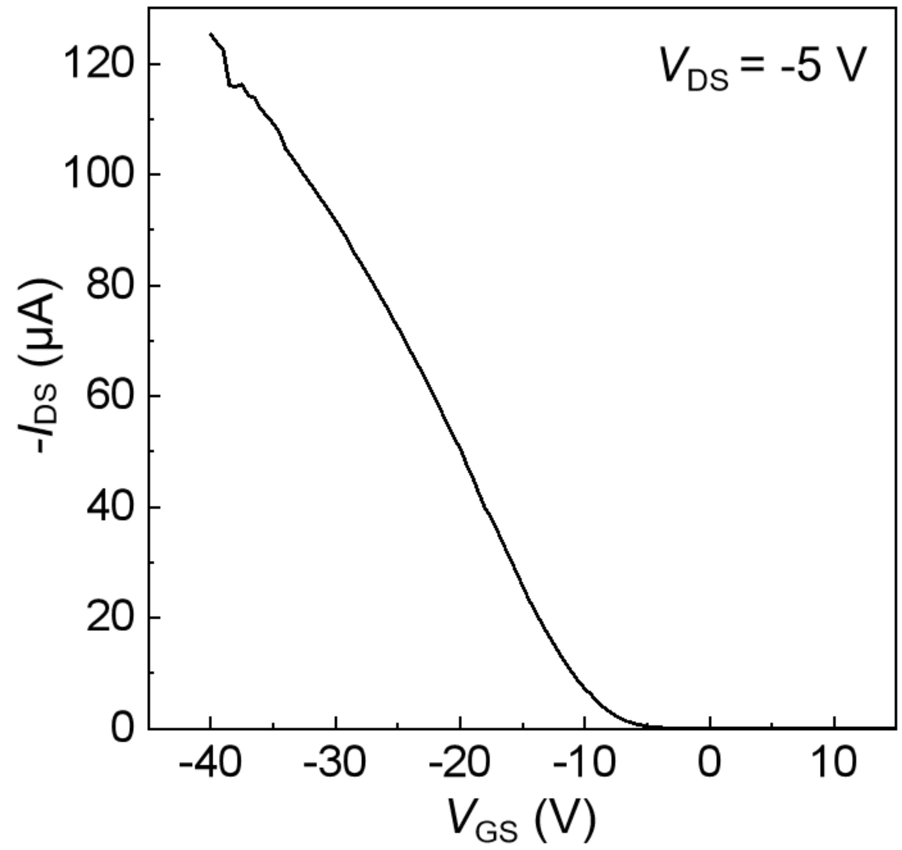

Figure S11. Transfer characteristic of the OTFT measured at linear region. The linear mobility estimated from the slope of the transfer curve is 15.3 $cm^2\ V^{-1}\ s^{-1}$.

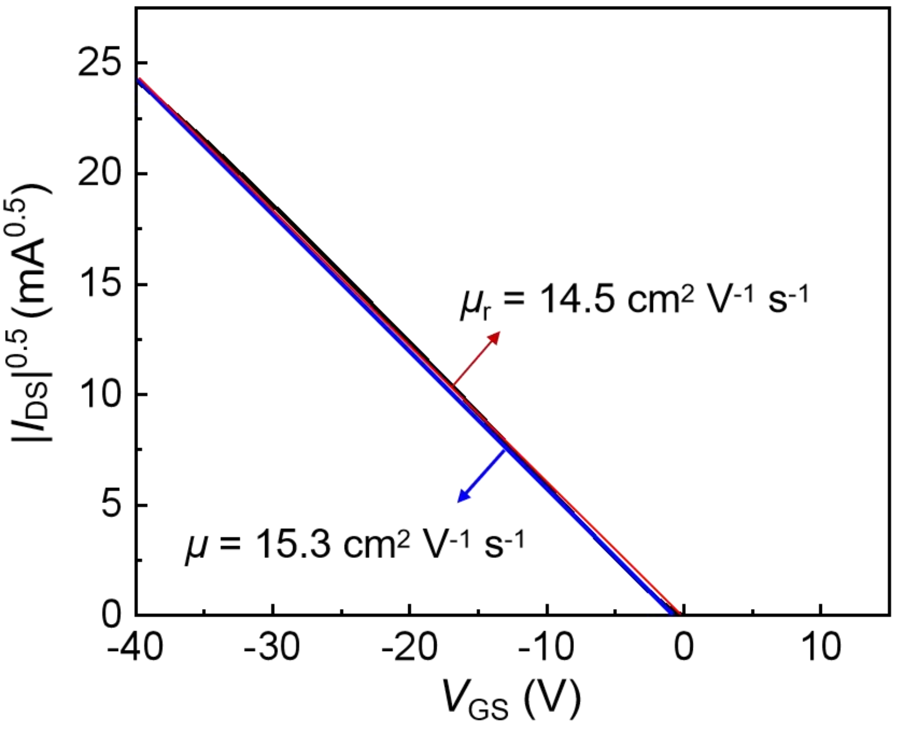

Figure S12. Transfer curve of the OTFT. The red line indicates the slope used in calculations of the μ_r. The blue line indicates the slope used in calculations of the μ.

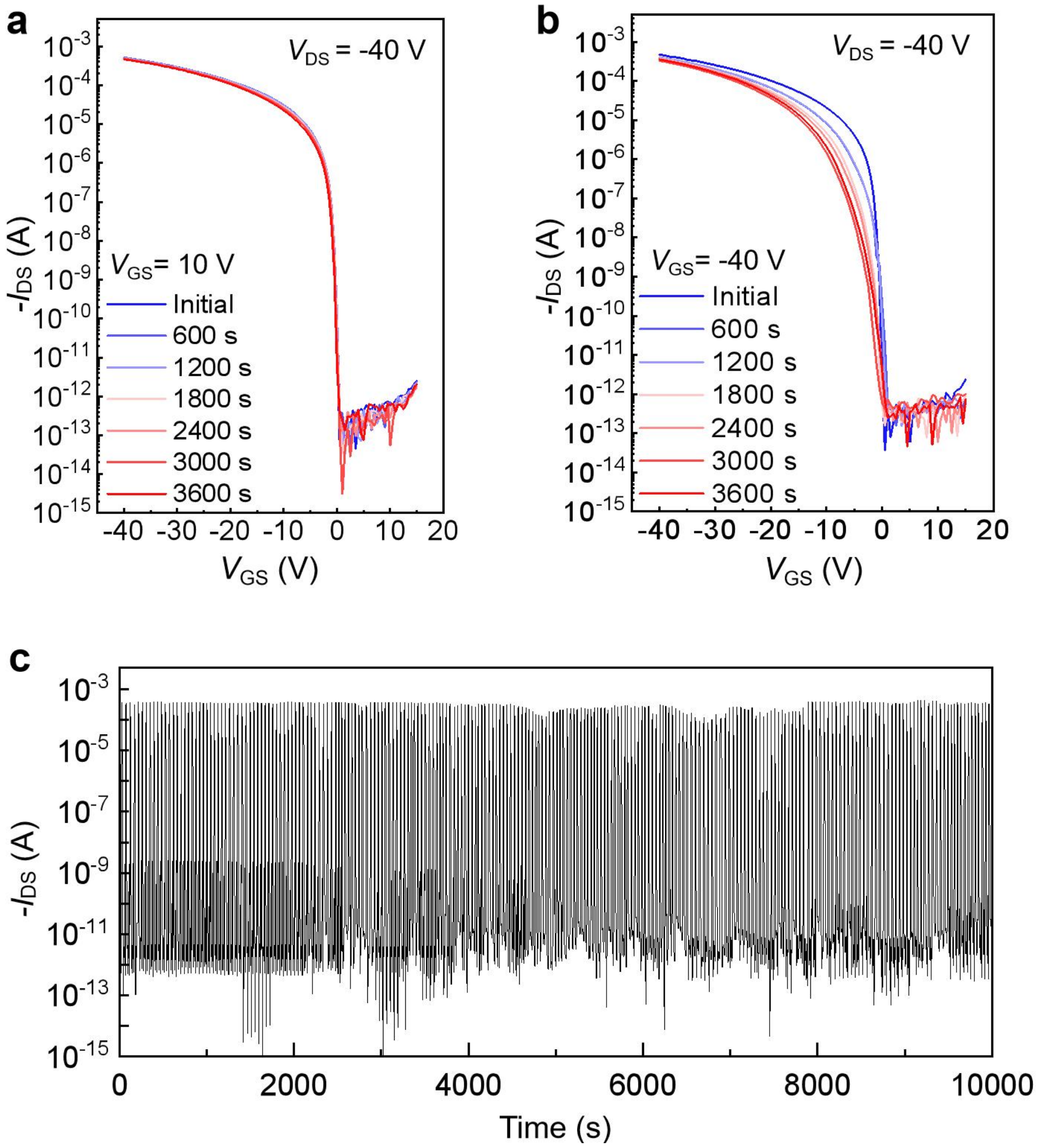

Figure S13. (a, b) Negative and positive bias stress testing results of the OTFT. (c) Drain current versus time plot of the OTFT under continuous bias conditions (V_{GS} = 10 V and -40 V).

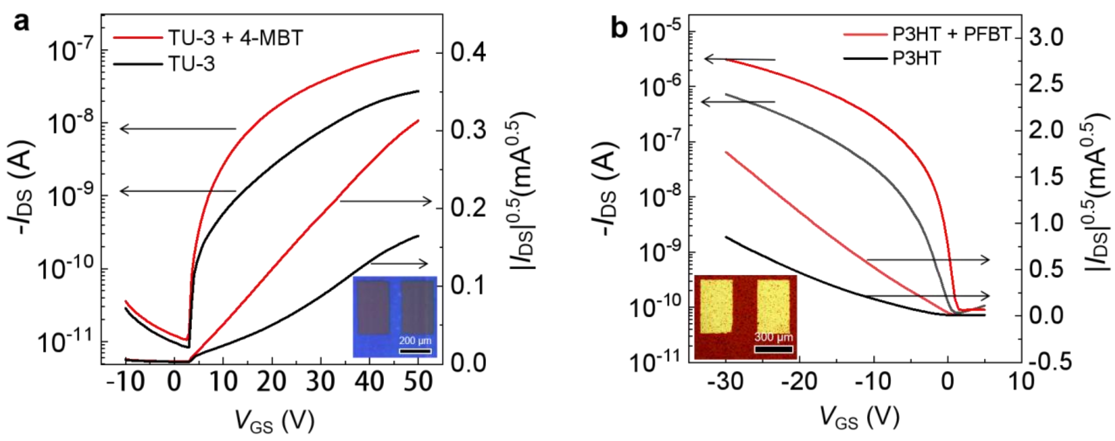

Figure S14. (a) Transfer curve of the TU-3 (black line) and TU-3 blend 4-MBT (red line) device. (b) Transfer curve of the P3HT (black line) and P3HT blend PFBT (red line) device.

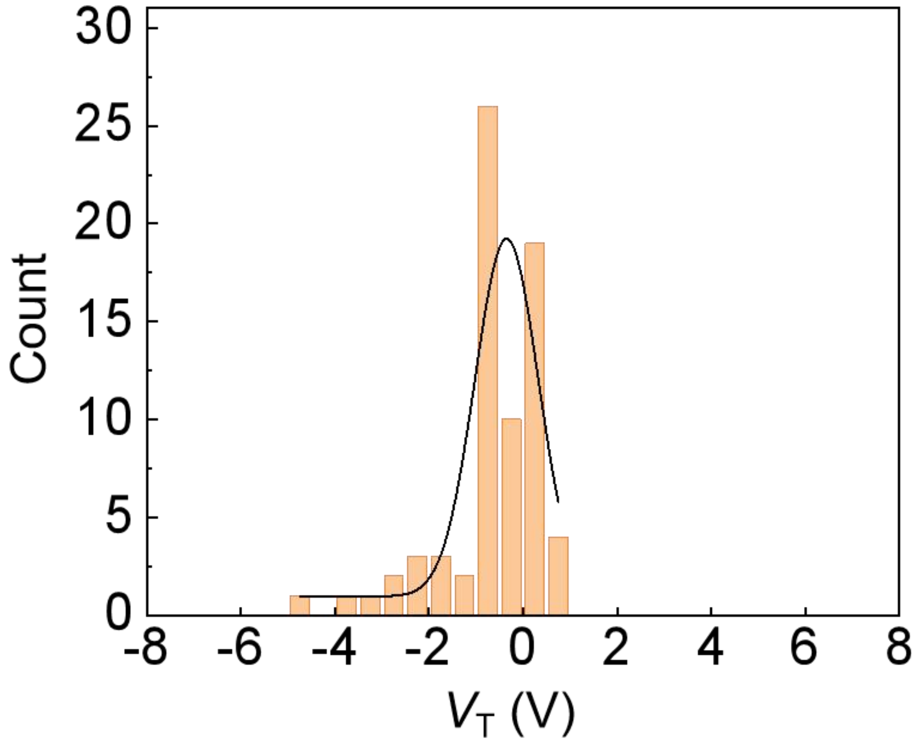

Figure S15. Statistical histogram of the V_T for the 72 devices.

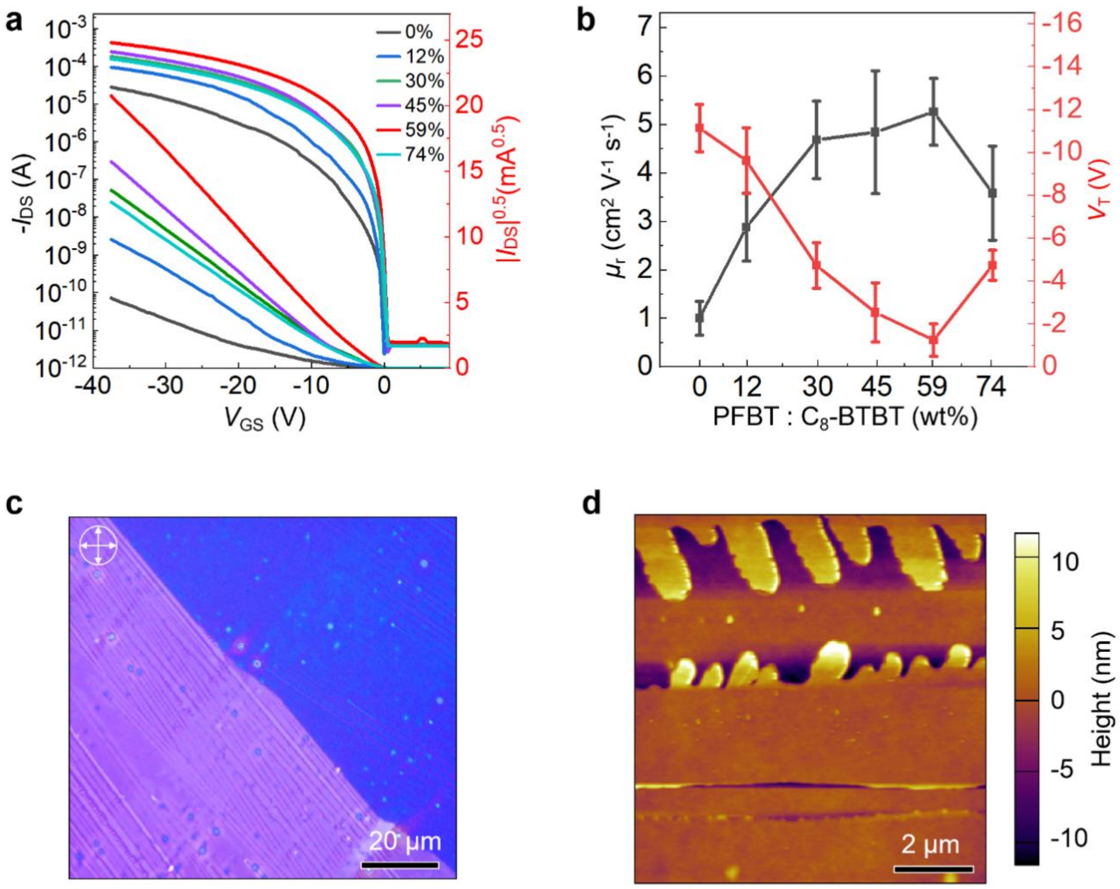

Figure S16. (a) Transfer curves of device with different weight percentage of PFBT in C_8-BTBT and PFBT blend solution. (b) Variation curves of V_T and μ_r with the weight percentage of PFBT in C_8-BTBT and PFBT blend solution. (c) CPOM images and the corresponding AFM morphologies (d) of C_8-BTBT: PFBT blend thin films where the mass ratio of PFBT to C_8-BTBT is 74 %.

The optimal PFBT: C_8-BTBT mass ratio (59 wt%) was determined through systematic device optimization. At this ratio, PFBT maximizes interfacial Ag–S bonding without perturbing the C_8-BTBT lattice, as confirmed by AFM and XRD. Excess PFBT disrupts crystallinity, highlighting the balance between interface engineering and structural integrity.

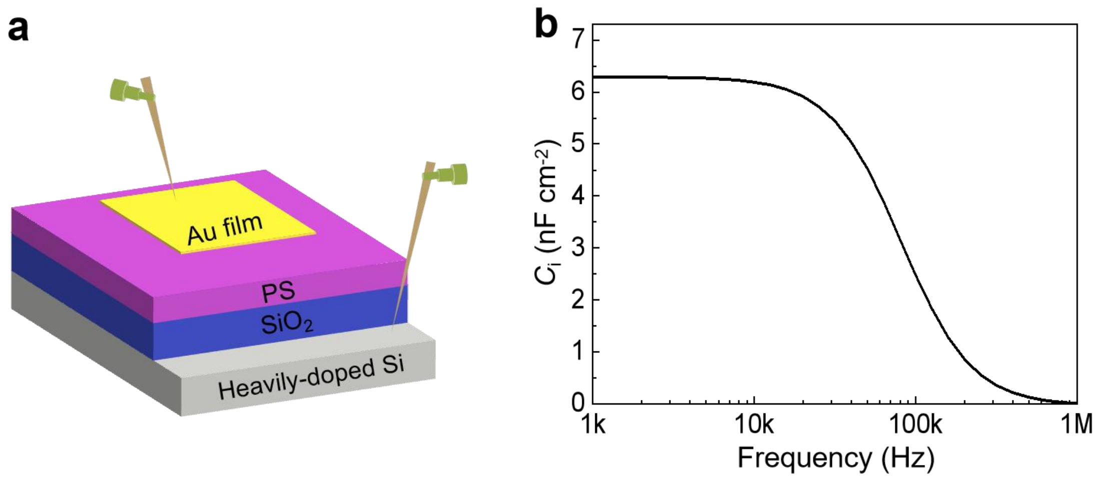

Figure S17. (a) Photograph of the sample for capacitance measurement. (b) Unit-area capacitance (C_i) of the PS/SiO_2 dielectrics at different frequencies.

Supporting tables

Table S1. Comparison of $R_c \cdot W$ at the same n_{2D}.

Channel materials	$R_c \cdot W$ (Ω·cm)	n_{2D} (× 10^{12} cm^{-2})	Ref.
**C_8-BTBT**	**79**	**2.2**	**This work**
**C_8-BTBT**	1936	2.2	Control device
**C_8-BTBT**	5000	1.3	25
	6250	1.0	
	7500	0.8	
**C_8-BTBT**	400000	1.0	26
	150000	1.3	
	20000	3.4	
**C_8-BTBT**	32500	1.0	26
	12500	1.3	
	500	5.1	
**C_{10}-DNTT**	186	5.5	14
	200	4.8	
	250	3.4	
	300	2.7	
	500	1.5	
**C_{10}-DNTT**	490	1.6	27
**C_{10}-DNTT**	60.4	7.9	28
**Ph-BTBT-C_{10}**	206	6.4	28
**Ph-BTBT-C_{10}**	2100	1.3	29
	1200	1.4	
**Ph-BTBT-C_{10}**	3800	0.8	29
**PCDTPT**	30900	0.7	30
**MoS_2**	60	3.1	31
	50	4.2	
	30	5.1	
**MoS_2**	200	2.1	31
	175	2.5	
	150	3.1	
	125	4.2	

**C_{60}**	4000000	0.8	32
	1760000	1.1	
	1350000	1.5	
	1200000	1.9	
**C_{60}**	1650000	0.6	32
	1000000	1.0	
	750000	1.3	
	520000	1.7	
**TIPS**	63300000	0.8	33
	52300000	1.2	
	35100000	1.6	
**TIPS**	10500000	0.8	33
	8100000	1.2	
	6500000	1.6	

Table S2. Comparison of μ_r and r with the previous reports.

Channel materials	μ_r (cm^2 V^{-1} s^{-1})	r	Ref.
C_8-BTBT	**16.1**	**0.998**	**This work**
	12.3	0.77	38
	6.9	0.69	39
	6.7	0.64	40
	1.3	0.75	41
	3.7	0.81	42
	5.5	0.56	43
	11.1	0.72	44
	0.03	0.76	45
	2.2	0.56	46
	2.5	0.28	47
	6.2	0.81	48
	1.2	0.51	49
	4.9	0.85	50
	8.1	0.81	11
	10.4	0.87	51
	14.5	0.90	10
	9.2	0.66	52
	0.7	0.16	53
	6.9	0.69	54
	1.3	0.25	55
C_{10}-DNTT	5.5	0.74	29
	4.3	0.64	31
Pentacene	1.3	0.60	29
DNTT	4.0	0.66	29
DPh-DNTT	6.0	0.69	29
TIPS	0.2	0.49	56
	0.4	0.56	
	1.6	0.64	
	2.1	0.64	
